# *Galleria mellonella*: The Versatile Host for Drug Discovery, In Vivo Toxicity Testing and Characterising Host-Pathogen Interactions

**DOI:** 10.3390/antibiotics10121545

**Published:** 2021-12-17

**Authors:** Magdalena Piatek, Gerard Sheehan, Kevin Kavanagh

**Affiliations:** SSPC Pharma Research Centre, Department of Biology, Maynooth University, Maynooth, W23 F2K8 Co. Kildare, Ireland; magdalena.piatek@mu.ie (M.P.); gerardsheehan1@gmail.com (G.S.)

**Keywords:** antimicrobial, *Galleria*, in vivo testing, in vivo toxicity

## Abstract

Larvae of the greater wax moth, *Galleria mellonella*, are a convenient in vivo model for assessing the activity and toxicity of antimicrobial agents and for studying the immune response to pathogens and provide results similar to those from mammals. *G. mellonella* larvae are now widely used in academia and industry and their use can assist in the identification and evaluation of novel antimicrobial agents. *Galleria* larvae are inexpensive to purchase and house, easy to inoculate, generate results within 24–48 h and their use is not restricted by legal or ethical considerations. This review will highlight how *Galleria* larvae can be used to assess the efficacy of novel antimicrobial therapies (photodynamic therapy, phage therapy, metal-based drugs, triazole-amino acid hybrids) and for determining the in vivo toxicity of compounds (e.g., food preservatives, ionic liquids) and/or solvents (polysorbate 80). In addition, the disease development processes are associated with a variety of pathogens (e.g., *Staphylococcus aureus*, *Listeria monocytogenes*, *Aspergillus fumigatus*, *Madurella mycotomatis*) in mammals are also present in *Galleria* larvae thus providing a simple in vivo model for characterising disease progression. The use of *Galleria* larvae offers many advantages and can lead to an acceleration in the development of novel antimicrobials and may be a prerequisite to mammalian testing.

## 1. Introduction

Insects possess a highly successful immune system that rapidly identifies pathogens and parasites and either kills them directly or immobilises them thus ensuring the survival of the host [1]. A wide range of structural and functional similarities exist between the insect immune response and the innate immune response of mammals [2,3] and, as a result, a wide variety of insects (*Galleria mellonella*, *Drosophila melanogaster*, *Manduca sexta*, *Bombyx mori*) is now used as in vivo models for assessing microbial virulence or for evaluating the in vivo efficacy and toxicity of antimicrobial compounds [4,5,6,7]. Larvae of the greater wax moth (*Galleria mellonella*) are widely used in academia and industry and in many cases generate results comparable to those that can be obtained using mammals [8,9,10]. Larvae have the advantage of being inexpensive to purchase and house, easy to manipulate, and being free from the legal and ethical restrictions that hinder vertebrate use [11]. This review will provide an insight into how *Galleria* larvae can be used to assess the in vivo efficacy and toxicity of novel antibacterial and antifungal agents and also to study disease development processes in vivo. As the number of applications of using *Galleria* larvae increase, their contribution to accelerating new drug development will increase.

## 2. *G. mellonella* Larvae as a Vehicle for In Vivo Antibacterial Drug Assessment

The multidrug resistant “ESKAPE” pathogens (*Enterococcus faecium*, *Staphylococcus aureus*, *Klebsiella pneumoniae*, *Acinetobacter baumannii*, *Pseudomonas aeruginosa* and *Enterobacter* spp.) are among a few that have rendered conventional antimicrobial therapies inactive, some of which are detailed below [12]. Due to increasing incidences of these resistant bacterial isolates to conventional antibiotics, there is an urgent need to develop novel antimicrobials with distinct modes of action. The use of *Galleria* larvae has contributed to this objective by allowing the accelerated in vivo assessment of potentially novel antimicrobial therapies. Phage therapy has proven effective against biofilm formation and aids penetration of antibiotics and/or cell lysis [13]. *G. mellonella* larvae have been used to assess a cocktail of phages CDHM 1, 2, 5 and 6 (multiplicity of infection = 10), as individual treatments and synergistically with vancomycin (64 µg/g). Prophylactical treatment offered the greatest level of protection against *Clostridium difficile* infection with 100% larval survival after 60 h. Further analysis indicated an increase in phage efficacy and reduction in bacterial load following prophylactic use of vancomycin [14].

Photodynamic therapy promoted the survival of *G. mellonella* larvae exposed to the periodontal pathogen *Porphyromonas gingivalis*. Inoculation of 1 × 10^6^ colony forming units (CFUs) of *P. gingivalis* per larva followed by 120 s of photodynamic therapy maintained 75% larval survival compared to untreated samples. This therapy also induced an immune response and a 2.62-fold increase in haemocyte (immune cell) density. Disruption of bacterial membranes enabling more efficient haemocyte-mediated phagocytosis was the suspected mode of action of this therapy [15].

**Figure 1:**
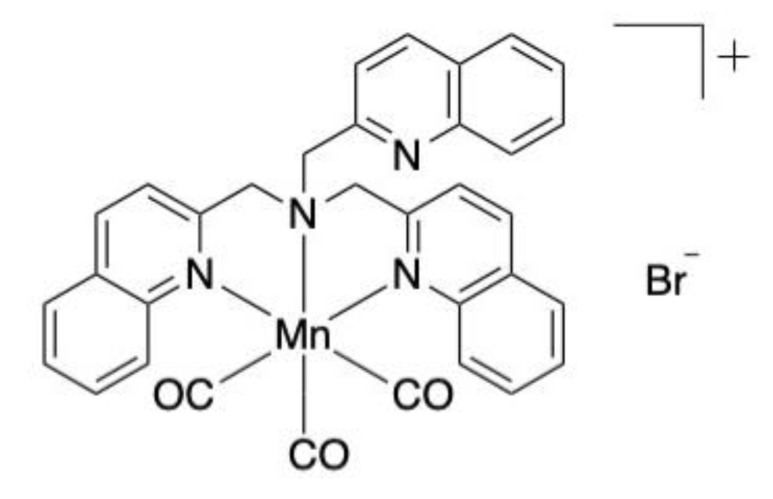
Chemical structure of [Mn(CO)_3_(tqa-κ^3^*N*)]Br evaluated in vivo. Adapted image [16].

Four novel manganese-based compounds have been assessed for their antibacterial activity through the weakening of bacterial membrane integrity: [Mn(bpqa-κ^3^*N*)(CO)_3_]Br, [Mn(bqpa-κ^3^*N*)(CO)_3_]Br, [Mn(CO)_3_(tqa-κ^3^*N*)]Br and [Mn(CO)_3_(tpa-κ^3^*N*)Br. Larvae infected with two multidrug-resistant clinical isolates of *Acinetobacter baumannii* and *Pseudomonas aeruginosa* were administered 5 mg/kg of [Mn(CO)_3_(tqa-κ^3^*N*)]Br (Figure 1) and observed at 24-h intervals. Approximately 80% of *A. baumannii*-infected larvae treated with the compound survived after 96 h in comparison with 50% of *P. aeruginosa* infected larvae [16].

Silver is a well-established component of many antimicrobial therapies used in wound dressings, creams and medical device coatings in healthcare settings [17]. *G. mellonella* larvae were employed to assess the effective dosing concentrations of silver nanoparticles (AgNPs). Toxicity evaluation deemed concentrations below 35 mg/kg suitable for in vivo use. Prophylactic treatment (25 mg/kg) resulted in 80% larval survival for up to four days while larvae infected with a highly virulent clinical isolate of *P. aeruginosa* developed disease symptoms and/or died in the first 24 h. Prophylactic treatment enhanced haemocyte production and maintained phenoloxidase activity similar to uninfected larvae. Lower levels of phenoloxidase and the presence of haemocyte nodules (which entrap and kill invading microbes) in response to AgNPs explained the reduced levels of melanisation of treated larvae [18,19].

**Figure 2:**
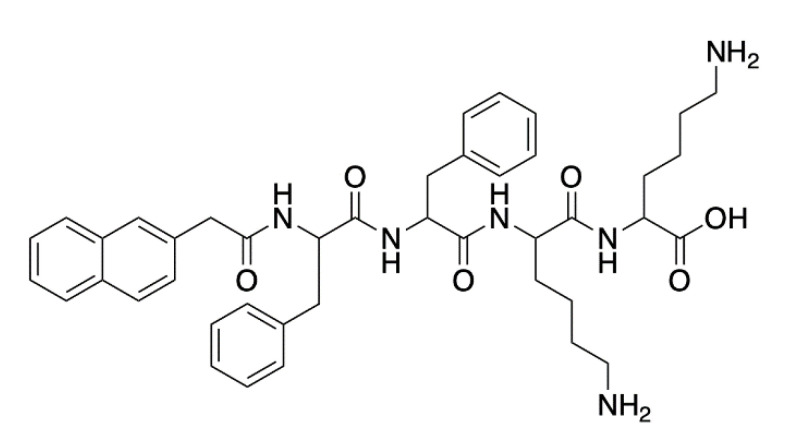
NapFFKK-OH hydrogel chemical structure. Adapted image [20].

*G. mellonella* larvae were administered the antimicrobial peptide-containing hydrogel (Naphthalene-2-ly)-acetyl-diphenylalanine-dilysine-OH (NapFFKK-OH) (Figure 2) via intra-hemocoel injection. Larvae injected with 0.5–2% (*w*/*v*) NapFFKK-OH maintained full survival up to 120 h, demonstrating more desirable results compared to in vitro assessment on murine fibroblast cells. In vivo testing revealed improved tolerability and the capacity to reduce bacterial loads after 24 and 72 h. The presence of viable *Staphylococcus epidermidis*, *Staphylococcus aureus*, *Escherichia coli* and *P. aeruginosa* decreased in a dose dependent manner, particularly in the case of *S. aureus* 72 h post-treatment (4.4 log_10_ CFU/mL reduction) [20].

## 3. Application of *G. mellonella* Larvae in Antifungal Drug Evaluation

**Figure 3:**
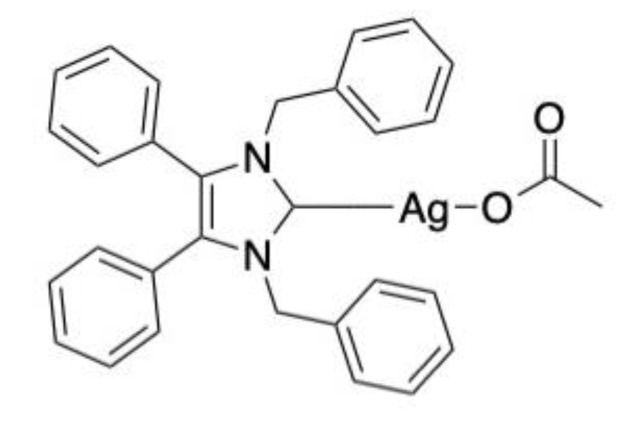
Chemical structure of SBC3.

Novel metal based antifungal therapies have been evaluated in *Galleria* larvae and results have shown potential applications in mammals. Rowan et al., (2009) assessed the antifungal efficacy of ([Ag_2_(mal)(phen)_3_], AgNO_3_ and 1,10-phenanthroline for their activity against *Candida albicans* in *G. mellonella* larvae. Larvae infected with 5 × 10^5^ *C. albicans* cells were treated prior to and post infection. Treatment one hour after infection ensured approximately 80% survival in all treatments after 24 h. This decreased to approximately 65% after 48 h and 30–40% after 72 h across all treatment types [21]. An alternative silver-based compound, 1,3-dibenzyl-4,5-diphenyl-imidazol-2-ylidene silver(I) acetate (SBC3) (Figure 3) has displayed potent antifungal activity, specifically against *C. albicans*. In vitro exposure to 25 μg/mL SBC3 inhibited *C. albicans* growth by 86.2 ± 1.42%. To verify this activity in vivo, larvae were firstly subjected to a lethal inoculum of *C. albicans* (1 × 10^6^ cells) that induced 100% mortality after 48 h. Treatment with 20 μL doses of 10, 100 and 250 μL/mL SBC3 four hours post-infection lead to 30 ± 5.77, 16.7 ± 6.66 and 13.3 ± 3.33% survival of larvae, respectively, after 72 h. Importantly, SBC3 alone did not elicit an immune response or toxic reaction in larvae [22].

Copper(II), Manganese(II), and Silver(I) 1,10-phenanthroline chelates displayed potent antifungal activity against *Candida haemulonii*. Eleven complexes containing copper, manganese and/or phen were administered one hour post-infection with 5 × 10^5^ *C. haemulonii* cells at concentrations of 5, 2.5, 1.25 and 0.625 μg/larva. Manganese-phen complexes appeared the most potent in reducing fungal load after 24 and 48 h. Prophylactic treatment 24 h in advance provided high levels of protection and reduced mortality by 55–100% after 24 h of infection. The previous evaluation identified non-toxic doses and increases in haemocyte density in response to some of the chelates [23].

Lim et al., (2018) identified ten compounds out of 800 with potent activity against *Madurella mycetomatis* in *G. mellonella* larvae. The top ten most active compounds that significantly enhanced larval survival include MMV-006357, -675968 and -022478. Out of five assessed fenarimol analogues, EPL-BS0178, -BS0495 and -BS1205 promoted survival by 36.7%, 24.1% and 19.2%, respectively. It has been suggested that the success of these fenarimols is attributed to their high polarity, and therefore, greater permeability and tissue delivery [24].

**Figure 4:**
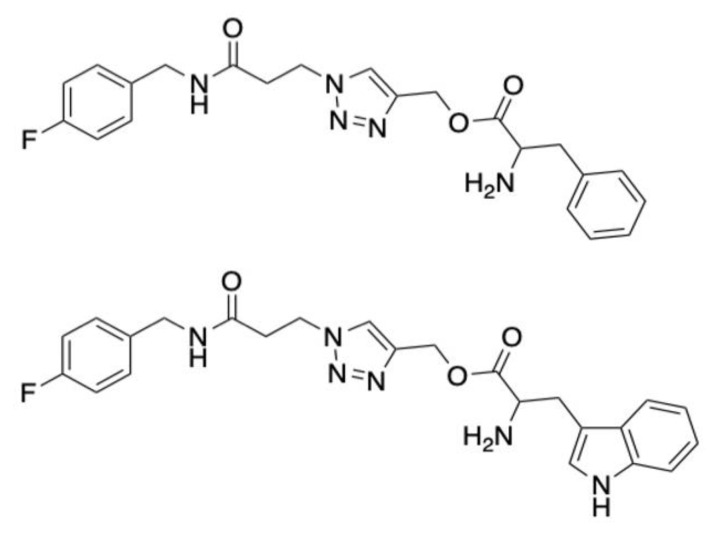
Chemical structure of Compounds 68 and 70. Adapted images [26].

Triazole-amino acid hybrids have shown strong promise as novel anticandidal treatments. These compounds, which bear an indole moiety, inhibit ergosterol biosynthesis and damage cell wall integrity. They are particularly effective in combination with current azoles [25,26]. A series of 24 compounds were screened to identify 1,2,3-triazoles consisting of phenylalanine and tryptophan tails as the most potent against fluconazole-resistant *C. albicans*. Compounds 68 and 70 (Figure 4) were non-toxic up 2.5 mg/mL in *G. mellonella* larvae and at this concentration could reduce *C. albicans* growth by approximately 50 and 70%, respectively. The compounds did not elicit an immune response as haemocyte density counts were unaltered [26].

**Figure 5:**
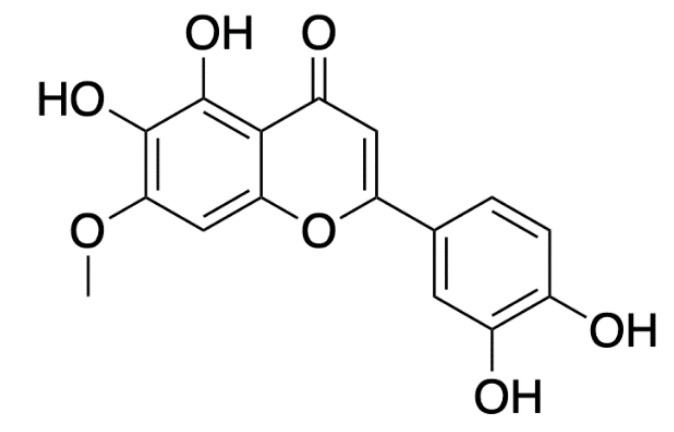
Chemical structure of pedalitin. Adapted image [28].

Pedalitin (Figure 5), a flavonoid extracted from *Pterogyne nitens* tree leaves, was injected alone and in combination with amphotericin B into *G. mellonella* larvae. No larvae survived after four days following infection with 1 × 10^6^ cells *C. neoformans* whereas combination therapy (amphotericin B, 0.3 mg/kg and pedalitin, 10 mg/kg) rescued approximately 80% and 60% of larvae at this time point and after seven days, respectively. Histopathology of larvae over three days supported these findings- untreated larvae experienced severe tissue damage in response to the yeast (1 × 10^6^ cells/mL) in contrast with individual (amphotericin B, 4 mg/kg; pedalitin 40 mg/kg) and combination therapy (amphotericin B, 0.3 mg/kg; pedalitin 10 mg/kg). The efficacy of pedalitin and/or amphotericin B in *G. mellonella* was comparable with murine results where combination therapy promoted 40% survival after 40 days. Infections are typically managed with fluconazole and amphotericin B however, pedalitin has shown potential as a less toxic alternative for cryptococcosis [27].

## 4. *G. mellonella* Larvae as a Novel In Vivo System for Toxicity Studies

*Galleria* larvae provide a fast and convenient means to assess in vivo toxicity and results show a strong correlation to those from mammalian systems. The Global Harmonising System of Classification and Labelling of Chemicals (GHS) classifies the acute toxicity of hazardous chemicals into five categories ranging from low toxicity (category 5) to high toxicity (category 1) and are based on the median lethal dose (LD_50_) (oral, dermal) and IC_50_ (inhalation) values [29]. A strong correlation between GHS classification and the *G. mellonella* larval response to several chemicals in comparison with 3T3 and NKH cell lines and in a murine model has been demonstrated [30]. The survival rates of larvae and LD_50_ values were determined following treatment with 19 soluble chemicals including disulfoton, cadmium chloride, phenol, citric acid, sodium hypochlorite and glycerol. The larval response to nine of these chemicals that were assigned to GHS category 5 correlated strongly with GHS classified measurements whereas only four out of nine tested on 3T3 and NHK cell lines matched GHS classification. Cell culture assays can exaggerate the toxicity of chemicals and so for this reason, in vivo analysis using *G. mellonella* larvae may provide a more accurate representation of low toxicity chemicals prior to animal testing [30].

A system of controlled chemical dosage based on the larval response after five days was devised [31]. *G. mellonella* larvae were initially administered 5 mg/kg and if ≥60% larvae survived after five days, new larvae were subjected to higher doses in a sequential manner up to a maximum dose of 2000 mg/kg to establish a toxic dose. Chemicals that induced 40% mortality following the starting dose were classified as GHS category 1 whilst those given 2000 mg/kg concentrations that resulted in 100% survival were deemed non-toxic. Calculated LD_50_ values for all 11 compounds were compared to known values according to the corresponding Material Safety Data Sheet (MSDS). Compounds such as doxorubicin showed the lowest LD_50_ value and proved most toxic to larvae matched values corresponding with mammalian models (5.5 mg/kg for *G. mellonella* versus 1.2 and 16 mg/kg in mice and rats, respectively). It is evident that *G. mellonella* larvae are strong candidates for acute toxicity testing and can help predict the toxic response in higher organisms thereby reducing the quantity of vertebrates required [31].

The toxicity of widely used food preservatives was examined to validate the suitability of *G. mellonella* as a toxicity screening model. Larvae were administered with potassium nitrate, potassium nitrite, potassium sorbate, sodium acetate, sodium benzoate, sodium chloride, sodium nitrate and sodium nitrite via force-feeding or intra-hemocoel injection and monitored over 48 h. All preservatives proved toxic to the host however, increased bioavailability post intra-hemocoel administration generated lower LD_50_ values in comparison with force-fed samples. Force feeding and intra-hemocoel injection LD_50_ values demonstrated a positive correlation with IC_50_ values calculated from human epithelial type 2 (HEp-2) cells after exposure to these preservatives. In addition, the relative toxicity corresponded with previously recorded results from murine models [32].

Ionic liquids are considered toxic, particularly those that consist of longer alkyl and hydroxyl chains. Imidazole-based ionic liquids are widely used in industry, however, they have demonstrated various levels of toxicity to enzymes, mammalian cells, and model organisms with regards to growth, viability and/or reproduction rates [33,34,35,36]. The survival of *G. mellonella* larvae was observed to estimate LD_50_ values of nine 1-alkyl-3-methylimidazolium chlorides with varied alkyl chains ranging from two to 18 carbon atoms. Chain lengths of C_8_ accounted for the highest level of toxicity with an LD_50_ of 11.7 μg/g. Thereafter, toxicity decreased with LD_50_ 69.2 μg/g for chain lengths of C_18_. The correlation between increased chain length and toxicity coincides with previous studies on other model organisms to a certain extent however, the high fat content in larvae and increasing lipophilicity of the compounds likely causes compound aggregation and reduced bioavailability in host tissues [37].

There is limited knowledge regarding optimal testing conditions in *G. mellonella* however, PBS, water, and DMSO (<20%) are the most widely used solvents for compound testing. Suay-García et al., (2019) analysed the tolerability of five aqueous solvents (acetic acid, DMSO, HCl, MeOH and NaOH) and four non-aqueous solvents (benzyl benzoate, ethyl oleate, isopropyl myristate and olive oil) in *G. mellonella*. All nine solvents were regarded as non-toxic and suitable for use in larvae except NaOH where concentrations above 0.5 M were lethal to 50% of larvae. The maximum concentrations of DMSO, MeOH and acetic acid were kept at 30%. Higher concentrations possess antimicrobial activity, and therefore, misrepresent drug activity in antimicrobial testing. Moreover, amino salicylic acid was selected as a compound to validate the delivery potential of these solvents. NaOH (0.5 M) successfully dissolved 50, 125 and 300 mg/kg doses of amino salicylic acid for larval administration whereas previous studies have eliminated its use due to solubility issues [38].

The safety of nanomaterial drug delivery systems must also be considered. For example, the size of nanoparticles and charge contribute to different levels of reactivity. Neutral and negative charges tend to be less reactive and hence less toxic in comparison to positively charged nanoparticles. *G. mellonella* larvae have been employed to assess the toxicity of lipid-core nanocapsules composed of polysorbate 80 (LNC-1), lecithin and polysorbate 80 (LNC-2) and lecithin, chitosan and polysorbate 80 (LCN-3). These lipid-core nanocapsules coated with micellar layers and a lipophilic crown provide a protective coating for drugs to prolong blood circulation and enhance delivery to specific targets. This study challenged larvae with formulations of LNC-1, 2 and 3 at concentrations ranging from 3.75 × 10^−14^ to 3.75 × 10^−10^ mol/kg via intra-hemocoel injection and monitored survival for 120 h. Survival rates were similar to those of DMSO and water control samples and showed no distinction between neutral, negative and positive nanocapsules. Moreover, Wistar rats treated with LNC-1,2 and 3 over 28 days demonstrated no signs of cardio-, nephro- or hepatotoxicity. There was also no evidence of tissue damage following blood and urine analyses or obvious signs of oxidative stress [39]. Thus, *G. mellonella* larvae were suggested as strong candidates for toxicity studies of nanomaterials [40].

## 5. Application of *G. mellonella* Larvae to Characterise Microbial Disease Development Processes In Vivo

**Figure 6:**
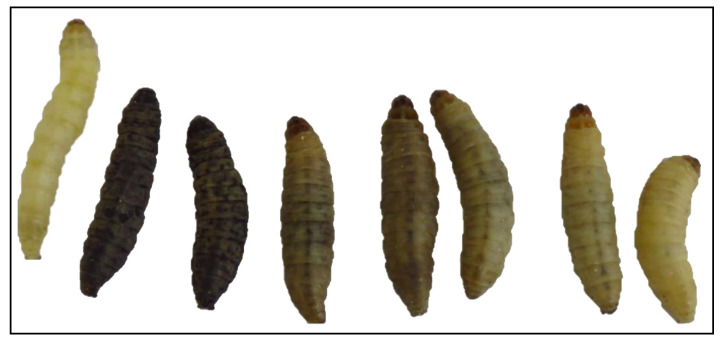
*G. mellonella* larvae with varying degrees of *S. aureus* infection 24 h post-inoculation. Larvae were injected with 20 μL of cell suspension in PBS and incubated at 37 °C for 24 h. *S. aureus* cell inoculum/larva from left to right: uninfected control larva (PBS only), 2 × 10^8^, 1 × 10^8^, 5 × 10^7^, 2 × 10^7^, 1.5 × 10^7^, 1 × 10^7^, 5 × 10^6^ cells/larva.

*Galleria* larvae are susceptible to infection by a wide range of bacterial and fungal pathogens. Recent work has highlighted how many of the pathologies associated with human infection by these pathogens are also visible in larvae as the infections develop. Infection of *G. mellonella* larvae with *Staphylococcus aureus* resulted in a dose dependent decrease in larval viability at 37 °C [41] (Figure 6). In mice, an inoculum of 10^8^ CFU/mouse resulted in 50% survival after 2 days and 0% survival after four days [42]. In neutrophil depleted mice, *S. aureus* can proliferate significantly within blood and skin samples [43]. By 48 h, widespread melanisation of insect haemolymph occurred possibly due to uncontrolled phenoloxidase activation as a result of bacterial replication [41]. Nodules produced in *G. mellonella* larvae during infection were similar in structure and composition to abscesses commonly found during *S. aureus* skin and soft tissue infection in humans [44]. *S. aureus* produces several molecules that contribute to abscess formation which recruit neutrophils, induce host cell lysis and the formation of the fibrin capsule surrounding the abscess [44]. Within 72 h staphylococci are localised within abscess at the centre of the lesions, enclosed by fibrin deposits, and surrounded by layers of immune cells [45,46].

*Listeria monocytogenes* displayed significant pathogenesis in *G. mellonella* larvae, and this was attributed to the expression of a range of virulence factors. Hexose phosphate transporter (uhpT), was essential for virulence, but factors such as PlcA appeared to be dispensable for virulence which was previously established in human umbilical vein endothelial cell (HUVEC) monolayers [47,48]. There was a large reduction in *L. monocytogenes* CFUs in larvae one hour post-infection suggesting the presence of effective constitutively expressed components of innate immune responses [47]. A range of AMPs such as gallerimycin, and lysozyme, were induced at six hours post-infection as assessed by qPCR and it was shown that inoculation of larvae with increasing concentrations of LPS augmented survival of larvae and the antimicrobial activity of haemolymph [47].

*L. monocytogenes* can induce the expression of gloverin and moricin AMPs during infection, target the central nervous system of *G. mellonella* larvae and relies on the same virulence factors required for human infection during infection of larvae [49]. Infection was associated with the formation of melanised nodules throughout the host as well as in the brains of larvae. Furthermore, *Listeria* infection resulted in developmental shifts and changes in gene expression encoding growth hormones [49]. Inhibitors of autophagy (rapamycin) or host cyclo-oxygenase (diclofenac) enhanced survival in larvae but also have anti-listerial activity in mammals [49,50,51]. *L. monocytogenes* can penetrate the blood-brain barrier in larvae and induce a brain specific immune response and infection which results in significant alterations in insect development [49].

*Klebsiella pneumoniae* infection of *G. mellonella* larvae showed similar features to *Klebsiella*-induced pneumonia in mammals. Infection of mice with *K. pneumoniae* is primarily characterised by cellular necrosis due to a high bacterial burden which results in a profound inflammatory response [52]. In *Galleria*, *Klebsiella* resulted in larval death due to rapid proliferation in haemolymph and a large and early (12 h post-infection) increase in phenoloxidase activity [52]. Interestingly, as with murine macrophages, *K. pneumoniae* avoided phagocytosis by *G. mellonella* haemocytes [53]. *K. pneumoniae* also produced a robust antimicrobial response by the increased expression of lysozyme, galiomycin, cecropin, gallerimycin and IMPI, key components of the insect immune response [52].

**Figure 7:**
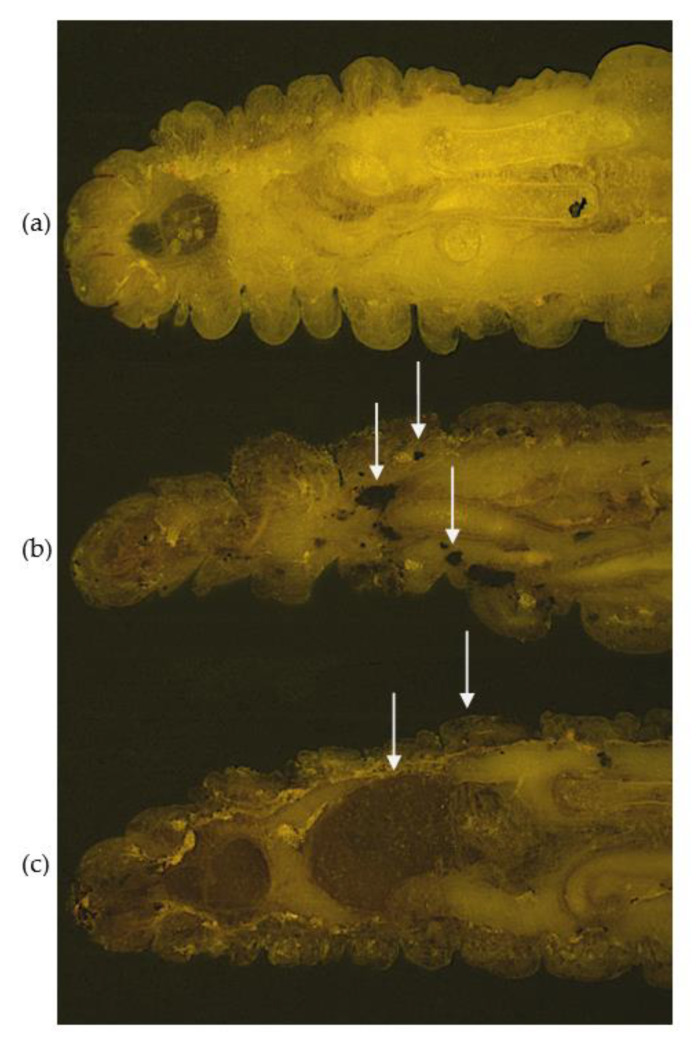
Cryo-images of *A. fumigatus* dissemination in the anterior sections of *G. mellonella* larvae after 24 h. (**a**) Uninfected larvae; larvae infected with *A. fumigatus* (**b**) 1 × 10^6^ and (**c**) 1 × 10^7^ viable conidia. Larvae were embedded in Cryo-imaging embedding compound and sectioned (10 μm) using Cryoviz^TM^ (Bioinvision Inc., Cleveland, OH, USA). White arrows depict fungal nodules/granulomas (**b**), melanised tissue and organs and cuticle melanisation (**c**).

*G. mellonella* larvae can be used to study *Aspergillus fumigatus* infection by comparing it to infection which occurs within the immunocompromised lung, or in those conditions which favour invasive aspergillosis [54]. A hallmark of invasive aspergillosis in the chronic granulomatous disease mouse model is the early germination of conidia and the formation of hyphae after 24 h with neutrophil infiltration and pyogranulomatous lesions surrounded by granulocytes [55]. During larval infection, there was dissemination of fungal material from the site of inoculation to distal sites and the resulting nodules consisted of granulocyte infiltration and encapsulation of *A. fumigatus* germinating conidia (Figure 7) [54]. Nodules showed similarities to granulomatous structures which are characteristic of invasive aspergillosis in the CGD murine model. The humoral immune response to *A. fumigatus* was examined and was associated with AMP production (gloverin, moricin, lysozyme, cecropin) and mediators of the phenoloxidase cascade (serpin-4B, prophenoloxidase activating enzyme 3 and prophenoloxidase activating factor 3) [54]. During the mammalian innate response to *A. fumigatus,* a range of antimicrobial peptides (defensins, cathelicidins) and proteins (lactoferrin, lysozyme) are produced and these are essential in curtailing early fungal establishment and growth [56,57]. Furthermore, the prophenoloxidase cascade is analogous to the mammalian complement protein cascade in terms of protein structure, function and mode of action [58,59]. During the later time point (24 h post-infection) proteins associated with tissue invasion (muscle protein 20 like protein), recognition and opsonization of fungal cells (hemolin, peptidoglycan recognition like protein B) and inhibition of fungal proteinases (insect metalloproteinase inhibitor (IMPI)) were increased in abundance [54,60,61]. *A. fumigatus* produces a variety of metalloproteinases most notably Asp f5/mep, a 42 kDa Zn/Mep, which possesses collagenlytic and elastinolytic activity and are important for immune cell recruitment in the murine lung [62,63].

*Madurella mycetomatis* is the dominant causative agent of eumycetoma, a chronic granulomatous type infection that is severely debilitating to its sufferers due to its occurrence at the extremities of the body. A key feature of mycetoma is the presence of grains inside the tissue and these grains may be formed as a defence mechanism by the fungus against the host immune system [64]. Grains consist of lipids, protein and melanin and the melanin is located on hyphal walls as thick layers. Furthermore, zinc, copper and calcium concentrations are significantly higher in *M. mycetomatis*-infected samples than controls which contribute to the formation of the grain cement matrix [65]. A proteomic approach was employed to characterise *M*. *mycetomatis* grain formation in *G. mellonella* larvae and map the processes leading to grain formation over time [66]. In the first stage the *G. mellonella* immune system deployed a range of cellular and humoral measures to contain and prevent the growth of *M. mycetomatis* consisting of proteins able to recognize fungal pathogen-associated molecular pattern (PAMPs) (β-glucan (PG-RPs) [67] and the opsonin lipopolysaccharide binding protein) [66]. *M. mycetomatis* responds by the production of vesicles associated proteins (e.g., GAPDH, enolase and fructose biosphosphate aldolase are known to bind constituents of the extracellular matrix) which were hypothesised to play a role in cement formation via transport of material into the extracellular space [66]. Furthermore, in the serum of eumycetoma patients, antibodies against *M. mycetomatis* fructose-bisphosphate aldolase and pyruvate kinase have been detected [68]. Immunohistochemistry demonstrated that fructose biosphosphate aldolase was expressed in the mammalian fungal grain [68].

## 6. Conclusions

*G. mellonella* is a highly versatile model organism that enables rapid in vivo assessment of the activity of antimicrobial compounds, the evaluation of the acute toxicity of a broad range of substances and the study of disease development processes which show strong similarities to infection processes in mammals. *G. mellonella* larvae are easy to inoculate and results are generated within 24–48 h. They also have the advantage of having a wide range of endpoints for each assay that gives additional information to inform subsequent in vivo mammalian testing. Despite these many advantages, *G. mellonella* larvae require further development as a model organism. For example, the generation of mutant strains has not yet become a possibility thus preventing genomic studies [69]. Although immune priming offers some degree of protection against re-infection in *Galleria* larvae, the lack of adaptive immunity in the form of antibodies, cytokines, dendritic cells and natural killer cells makes it difficult to ascertain the mammalian immune response [70,71]. Lastly, discrepancies among experimental procedures and larval stock supplies warrant a more standardised approach [69]. While there are some limitations, interest in the use of *G. mellonella* larvae is increasing as they provide a rapid, easy to use in vivo system which can expedite antimicrobial drug development.
